# Incidence and Characteristics of Acute Kidney Injury in Severe Diabetic Ketoacidosis

**DOI:** 10.1371/journal.pone.0110925

**Published:** 2014-10-22

**Authors:** Jean-Christophe Orban, Eve-Marie Maizière, Anis Ghaddab, Emmanuel Van Obberghen, Carole Ichai

**Affiliations:** 1 Réanimation médico-chirurgicale, Hôpital Saint-Roch, CHU de Nice, Nice, France; 2 IRCAN, Faculté de Médecine, Université de Nice Sophia-Antipolis, Nice, France; 3 Laboratoire de Biochimie, CHU de Nice, Nice, France; University of Leicester, United Kingdom

## Abstract

**Aims:**

Acute kidney injury is a classical complication of diabetic ketoacidosis. However, to the best of our knowledge, no study has reported the incidence and characteristics of acute kidney injury since the consensus definition was issued.

**Methods:**

Retrospective study of all cases of severe diabetic ketoacidosis hospitalised consecutively in a medical surgical tertiary ICU during 10 years. Patients were dichotomised in with AKI and without AKI on admission according to the RIFLE classification. Clinical and biological parameters were compared in these populations. Risk factors of presenting AKI on admission were searched for.

**Results:**

Ninety-four patients were included in the study. According to the RIFLE criteria, 47 patients (50%) presented acute kidney injury on admission; most of them were in the risk class (51%). At 12 and 24 hours, the percentage of AKI patients decreased to 26% and 27% respectively. During the first 24 hours, 3 patients needed renal replacement therapy. Acute renal failure on admission was associated with a more advanced age, SAPS 2 and more severe biological impairments. Treatments were not different between groups except for insulin infusion. Logistic regression found 3 risk factors of presenting AKI on admission: age (odds ratio 1.060 [1.020–1.100], p<0.01), blood glucose (odds ratio 1.101 [1.039–1.166], p<0.01) and serum protein (odds ratio 0.928 [0.865–0.997], p = 0.04).

**Conclusions:**

Acute kidney injury is frequently associated with severe diabetic ketoacidosis on admission in ICU. Most of the time, this AKI is transient and characterised by a volume-responsiveness to fluid infusion used in DKA treatment. Age, blood glucose and serum protein are associated to the occurrence of AKI on ICU admission.

## Introduction

The incidence of diabetes mellitus is increasing worldwide affecting both types of the disease. The most frequent acute diabetic complications are hyperglycemic crises, namely diabetic ketoacidosis (DKA) and hyperosmolar hyperglycemic state. Diabetic ketoacidosis results from an absolute insulin deficiency. Classical presentation associates a triad of uncontrolled hyperglycemia, metabolic acidosis and high ketone bodies concentration. Similarly to diabetes, the incidence of DKA increases over time [1], [2]. This may be a life-threatening condition due to severe clinical and biological impairments and treatment associated complications (cerebral edema, acute respiratory distress syndrome, hypokalaemia, hypophosphatemia). However, mortality is low and most of the time, death is related to the precipitating factor [3]–[6]. For this reason, admission of these patients in ICU is still debated. A grading system for severity of DKA was described previously [7]. Patients presenting the most severe grades or common severity criteria were considered for ICU admission. However this grading system is not recommended for clinical practice, resulting in wide variations in ICU utilisation for DKA, depending on the national practices, the number of DKA admitted in the units and the severity of the clinical status [8]. Interestingly, in the absence of randomised trials, there are no data showing any impact of the level of care on DKA mortality. To help clinicians, guidelines for DKA management are published and updated by the American Diabetic Association [6]. Their effect on clinical outcome is unclear as compliance to guidelines is poor in diabetes care and ICU [9], [10]. However, implementation of a local mandatory protocol seems more efficient to decrease ICU and length of hospital stay [11].

DKA is associated to numerous acid-base, hydration and electrolytes derangements [12]–[14]. Acute kidney injury (AKI) is deduced from admission laboratory tests and associated to the severity of the hyperglycemic crisis [15]. In this setting AKI proceeds mainly from hypovolemia due to glucose-induced osmotic polyuria and sometimes emesis. However, for years, non-consensual definitions were used, making it difficult to obtain an accurate evaluation of the epidemiology of AKI. Nowadays, based on the RIFLE classification [16], it is possible to compare the incidence and severity of AKI across different populations and settings. Acute renal dysfunction remains poorly reported in patients with severe DKA [17]. Therefore, the aim of our study was to describe the characteristics of AKI based on the RIFLE classification in severe diabetic ketoacidosis admitted in ICU, to compare patients with or without AKI and to find risk factors of AKI on admission.

## Materials and Methods

### 1. Patients selection and study design

Adult patients admitted consecutively in our medical surgical tertiary ICU for diabetic ketoacidosis between 2003 and 2013 were retrospectively reviewed. The hospital ethic committee (Comité de Protection des Personnes Sud Méditerranée V) approved this study and waived the need for patient's consent. Patients’ records were anonymized before analysis. The diagnosis of severe ketoacidosis was defined by the association of high blood glucose (>250 mg/dl), urine ketone and metabolic acidosis with a pH <7.30.

### 2. Patients’ management

Patients were treated according to the same local protocol of care adapted from the ADA guidelines [6], [18]. Briefly patients diagnosed DKA in the Emergency Department were given 500 mL to 1000 mL of normal saline and then an intravenous bolus of 0.1 UI/kg of regular insulin. Then, they were transferred to our ICU where a continuous infusion of insulin at a rate of 0.1 UI/kg/h was started. The rate was changed according to blood glucose measured every hour. Normal saline was infused according to clinical evaluation and/or haemodynamic monitoring. When blood glucose reached 2.5 g/L, insulin infusion was decreased while glucose infusion was added. Urine ketone was monitored every hour until disappearance. Potassium and phosphate supplementations were based on results of regular blood samplings.

### 3. Study protocol

We highlighted the following clinical data: age, sex, type of diabetes mellitus, presumed cause of DKA, body temperature, Glasgow Coma Scale score, haemodynamic or respiratory failure, time of ketosis resolution, Simplified Acute Physiology Score 2 (SAPS 2) and vital status at ICU discharge. Most biological parameters were collected on admission and after 12 and 24 hours: pH, bicarbonate, sodium, potassium, chloride, blood glucose, phosphate, creatinine, blood urea nitrogen (BUN) and calculated plasma osmolarity (natremia *2+ blood glucose+BUN). Organ failure during the first 24 hours was collected: haemodynamic failure defined as hypotension needing vasopressor use or fluid infusion and respiratory failure defined as the need for mechanical ventilation. Major treatments including insulin, type and amount of fluids and electrolytes supplementation were also collected. The frequency of blood sampling was protocol driven until resolution of ketosis assessed by disappearance of urine ketone. For this reason, data from patients discharged before 12 or 24 hours are missing at these time points.

The RIFLE classification was used to evaluate kidney injury on admission and on subsequent times [16]. For most patients, baseline renal function and serum creatinine were not known on admission. In these cases, serum creatinine levels were searched at the occasion of subsequent biological evaluation. When no indication on baseline renal function was available, we estimated the renal function according to published formula [16].

### 4. Study endpoints

The primary study endpoint was the comparison of the different clinical, biological and treatment parameters between patients according to the presence of acute kidney injury on admission in ICU. Acute kidney injury was assessed by the RIFLE classification.

Secondary study endpoints included the RIFLE classification of patients on admission, after 12 and 24 hours; and the search of risk factors of presenting AKI on admission in ICU.

### 5. Statistical analysis

Data are expressed as median and interquartile range or number and percentage. According to RIFLE classification, patients were dichotomised in with AKI and without AKI. We compared the baseline characteristics and clinically relevant variables of patients with and without AKI using Mann-Whitney U and chi-square tests, as appropriate. To identify the predictors of AKI on admission, we carried out a stepwise logistic regression model using the following variables: age, Glasgow Coma Scale, body temperature, SAPS 2 score, hemodynamic failure, blood glucose, serum chloride and protein. Correlations were made with a single regression test. We considered p<0.05 as statistically significant. All analyses were performed using XLSTAT version 2013.2.01 (Addinsoft, New York, NY).

## Results

Over 10 years (from 2003 to 2013), 94 patients were admitted in our ICU for diabetic ketoacidosis. Most of them presented type 1 diabetes mellitus (90%). Demographic data and biological parameters are reported in table 1. The causes of DKA were interruption of diabetes treatment (41%), infection (17%), newly-onset type 1 diabetes (16%), unknown (15%) and miscellaneous events (11%). Haemodynamic failure was present in 13% and respiratory failure requiring orotracheal intubation in 9%. Three patients died in ICU (3%), due to a septic shock in all cases.

**Table 1:** Proportions of patients with normal renal function and AKI at the three times of the study.

	Admission (n = 94)	H12 (n = 80)	H24 (n = 48)
**No AKI**	47 (50%)	59 (74%)	35 (73%)
**AKI**	47 (50%)	21 (26%)	13 (27%)
** Risk**	24 (51%)	10 (48%)	5 (38%)
** Injury**	13 (28%)	5 (24%)	3 (23%)
** Failure**	10 (21%)	6 (28%)	5 (38%)

In the AKI patients, RIFLE categories are reported as numbers and percentages of AKI patients.

Six patients of the population had chronic renal disease. According to RIFLE criteria, 47 patients (50%) presented acute kidney injury on admission: risk (51%), injury (28%) and failure (21%). Of these 47 patients, 5 had previously known chronic kidney disease. At 12 hours, the number of AKI patients decreased to 21 (26%, n = 80) with similar proportions of risk (48%), injury (24%) and failure (28%). After 24 hours, 13 patients (27%, n = 48) still presented AKI: risk (38%), injury (23%) and failure (38%). These results are reported in the table 1. The proportion of patients without AKI increased significantly from admission (50%) to 24 h (73%); p<0.01). During the first 24 hours, 3 patients needed renal replacement therapy.

Acute kidney injury on admission was associated with a more advanced age and SAPS 2. These patients exhibited more severe abnormal laboratory tests results as reported in table 2. Logistic regression was used to examine possible predictors of AKI on admission. Variables examined included age, Glasgow Coma Scale, body temperature, SAPS 2 score, hemodynamic failure, blood glucose, serum chloride and protein. The results of the logistic regression are reported in the table 3. The only predictors of AKI were age, blood glucose and serum protein.

**Table 2:** Baseline clinical and biological characteristics of the patient population with focus on renal function.

	All patients	No AKI	AKI	p value
**Age (years)**	44 [26–57]	28 [20–44]	52 [43–63]	p<0.001
**Glasgow coma score**	15 [14]–[15]	15 [15–15]	14 [12]–[15]	p<0.001
**Body temperature (°C)**	37 [36.2–37.4]	37.2 [36.6–38.0]	36.5 [35.0–37.0]	p<0.001
**SAPS 2**	28 [21–36]	24 [18]–[33]	31 [23–38]	p = 0.03
**Haemodynamic failure**	12 (13)	10 (10)	2 (2)	p = 0.015
**pH**	7.16 [7.01–7.26]	7.17 [7.06–7.27]	7.13 [6.96–7.26]	p = 0.14
**Bicarbonate (mmol/L)**	6.5 [3.0–10.5]	7.0 [3.7–10.0]	6.0 [3.0–11.0]	p = 0.52
**Glucose (mmol/L)**	29.5 [17.3–38.7]	21.0 [13.6–31.3]	38.0 [28.3–47.1]	p<0.001
**Sodium (mmol/L)**	135 [131–138]	135 [132–137]	135 [130–139]	p = 0.96
**Potassium (mmol/L)**	4.2 [3.7–4.8]	4.2 [3.6–4.7]	4.2 [3.7–5.0]	p = 0.31
**Chloride (mmol/L)**	97 [92–102]	99 [95–103]	95 [90–101]	p = 0.03
**Creatinine (µmol/L)**	124 [91–177]	91 [78–107]	173 [137–254]	p<0.001
**BUN (mmol/L)**	11.1 [6.8–16.3]	6.9 [4.7–10.1]	16.2 [13.1–23.9]	p<0.001
**Protein (g/L)**	71 [62–78]	75 [65–79]	69 [59–75]	p = 0.01
**Phosphate (mmol/L)**	1.18 [0.69–1.83]	0.89 [0.50–1.16]	1.64 [1.21–2.42]	p<0.001
**Anion gap (mmol/L)**	30 [24–36]	28 [23]–[33]	32 [27–37]	p = 0.02
**Osmolarity (mosm/L)**	314 [301–330]	303 [297–315]	329 [316–340]	p<0.001

Data are expressed as median and interquartile range except for haemodynamic failure expressed as number and percentage.

**Table 3:** Multiple logistic regression model with AKI on admission as the dependent variable.

Variable	Odds-ratio (95% CI)	p value
Age	1.060 (1.020–1.100)	0.003
Glasgow coma score	0.978 (0.692–1.384)	0.90
Body temperature	0.681 (0.412–1.126)	0.13
SAPS 2	1.003 (0.958–1.051)	0.89
Hemodynamic failure	0.167 (0.005–5.861)	0.33
Glucose	1.101 (1.039–1.166)	0.001
Chloride	0.962 (0.875–1.058)	0.43
Protein	0.928 (0.865–0.997)	0.04

Treatments were not different between groups except for insulin infusion (Table 4). There was no relationship between blood glucose on admission and the amount of insulin infused in the normal renal function group (R^2^ = 0.036, p = 0.2) whereas a correlation was found in the AKI group (R^2^ = 0.149, p = 0.007).

**Table 4:** Treatments administered in the population and according to the renal function.

	All patients	No AKI	AKI	
**Fluids (L)**	4 [3.0–5.0]	4 [3.5–4.7]	4 [3–5.5]	p = 0.58
**Insulin (UI)**	97 [70–144]	86 [59–118]	122 [85–160]	p = 0.005
**Potassium (g)**	7.0 [4.0–12.0]	7.0 [3.6–11.6]	7.5 [4.3–12.5]	p = 0.60
**Phosphate (g)**	6.0 [3.0–8.0]	6.0 [3.0–8.0]	6.0 [3.0–8.5]	p = 0.79
**Duration of resolution (h)**	17 [12]–[24]	16 [12]–[24]	18 [12]–[24]	p = 0.65

Data are expressed as median and interquartile range.

## Discussion

Our study reports for the first time to the best of our knowledge the incidence and severity of acute kidney injury in patients with severe diabetic ketoacidosis in ICU. Half of them presented AKI on admission but most of them were in the less severe RIFLE category. Elevated creatinine is reported to be a usual feature of hyperglycemic crises [6], [15] and more pronounced in hyperglycemic hyperosmolar state compared to DKA. However, no data on the incidence of AKI have so far been reported since the consensus definition was published.

The cause of AKI in hyperglycemic crisis is assumed to be “pre-renal”. Kidney hypoperfusion results from hypovolemia due to osmotic polyuria and sometimes gastrointestinal losses. However, accurate assessment of volemia in these patients is difficult, as invasive monitoring is not routinely set up. Although they are less reliable, only markers of volemia are available in our study. Surprisingly, haemodynamic instability was less frequent in AKI patients and the amount of fluid infused during the first day (surrogate of fluid deficit and hypovolemia) was not different. Moreover, serum protein, a sign of extracellular dehydration was lower in the AKI patients. These data fall short to support with confidence the assumption that in this setting, AKI results from a greater fluid deficit or hypovolemia. One possible explanation might be the difference of age between groups, as AKI patients are older. This could explain partly the development of AKI in our population as renal function declines with age [19].

Almost 50% of AKI patients recovered a normal renal function in less than 24 hours. Similar findings were reported in a subgroup of AKI patients where renal dysfunction returned to normal in 24 hours [20]. This particular form of pre-renal AKI is named transient azotemia. It is defined by an AKI recovering in less than 3 days [20]. However, the definition does not take into account the aetiology and the treatment of AKI. Recently a new concept called “volume-responsive AKI” [21] has been reported for renal functional impairment that can be improved by fluid administration. Clearly this definition fits the majority of AKI patients of our population as massive amounts of fluids were given during the first 24 hours.

In general AKI in critically ill patients is considered an independent risk factor for an increased morbimortality [20], [22]. However, our study reported a low mortality as previously described in DKA patients [3], [6], [7], [11]. The type of AKI, transient azotemia, partly explains this difference. This condition is associated with a better prognosis than other types of AKI such as acute tubular necrosis [20]. Moreover, less severe RIFLE classes are more frequent in transient azotemia. Accordingly, half of our AKI patients were classified as “risk” on admission in ICU.

Despite the same severity according to acid base status, AKI patients presented higher blood glucose on admission in ICU. This result could be explained by at least the two following causes. First, AKI seems associated to inflammation and insulin resistance in critically ill patients [23], [24]. Second, renal failure could be associated to a lower glycosuria, which is an effective way to limit hyperglycemia. When blood glucose remains within physiological levels, it is completely reabsorbed by the kidney. This process is concomitant of sodium reabsorption and involves mainly 2 co-transporters, sodium-glucose linked transporter (SGLT) 1 and 2. In diabetes, glucose absorption by SGLT 1 and 2 is increased [25]. However, when hyperglycemia exceeds the threshold value of glucose reabsorption, glycosuria appears to be limiting hyperglycemia. In AKI patients, renal perfusion is compromised and, by this way, could decrease glucose urinary losses and consequently combat hyperglycemia.

Recently the influence of hyperchloremia on renal function during ICU stay has emerged [26]. However, this relation was not found on patient admission as serum chloride was not different between AKI and non-AKI patients in a general ICU population [27]. Surprisingly, our study reports a lower serum chloride in AKI patients. This result can be explained by concomitant excretion of ketone bodies and chloride retention [28]. During AKI, the altered renal perfusion could decrease ketone bodies excretion and chloride retention.

In our population, AKI patients exhibited higher serum phosphate. Hyperphosphatemia is frequently found in AKI [29] and DKA patients [30]. In DKA, this results from intracellular to extracellular shifts due mainly to insulin deficiency whereas compromised renal perfusion leads to hyperphosphatemia in AKI. However, despite higher serum phosphate in AKI patients, the amount of phosphate infused during the first day was not different. This could be explained by a higher amount of insulin infused in the AKI patients and the improvement of renal function. Thus, even if patients exhibit higher serum phosphate, they will need supplementation.

Our study found 3 risk factors for AKI on admission in severe DKA patients: age, blood glucose and serum protein. In clinical practice, these variables are not prognosis factors nor included in the different DKA severity scores. But, they could help to take into account the particularities of AKI patients in this setting. Initial treatment of AKI is based on correction of hypovolemia by fluid infusion [16], [31]. But, excessive fluid infusion can lead to pulmonary edema, especially in case of oliguria. This is particularly true in aged diabetic patients who present more often diastolic dysfunction [32], [33]. These patients need more aggressive care and invasive monitoring that is best achieved in ICU. So, these risk factors associated to described severity grading score [7] could help select high-risk patients to be considered for ICU admission.

While offering novel insights, some aspects of our study have to be interpreted with caution. A first possible confounding effect is that our patients were cared in the Emergency Department before their admission in our ICU. However, in general they had the same treatment (fluid infusion of 1000 ml of normal saline and an intravenous bolus of 0.1 unit/kg of regular insulin). The second limitation is the lack of protocol in the Emergency Department for transfer of DKA in ICU. However, according to the median value of bicarbonate, the patients could be considered severe DKA [6] and needed admission to ICU [7]. The last caution note on our study concerns the fact that we did not compare the AKI population according to the RIFLE classification. Although differences have been reported in large populations [34], we chose not to compare the different classes due to the low number of patients.

## Conclusions

Acute kidney injury is frequently associated to severe diabetic ketoacidosis on admission in ICU. Most of the time, this AKI is transient and characterised by a volume-responsiveness to fluid infusion used in DKA treatment. AKI patients exhibit more severe clinical impairments and abnormal laboratory tests results but only age, blood glucose and serum protein are independently associated to the occurrence of AKI on ICU admission. Larger studies are needed to assess the influence of AKI class on outcome.
